# Analysis of the Precision of Variable Flip Angle *T*_1_ Mapping with Emphasis on the Noise Propagated from RF Transmit Field Maps

**DOI:** 10.3389/fnins.2017.00106

**Published:** 2017-03-09

**Authors:** Yoojin Lee, Martina F. Callaghan, Zoltan Nagy

**Affiliations:** ^1^Laboratory for Social and Neural Systems Research, University of ZürichZürich, Switzerland; ^2^Department of Information Technology and Electrical Engineering, Institute of Biomedical Engineering, ETH ZürichZürich, Switzerland; ^3^Wellcome Trust Centre for Neuroimaging, UCL Institute of Neurology, University College LondonLondon, UK

**Keywords:** *B*_1_^+^ map, *T*_1_ map, error propagation, uncertainty, precision, variable flip angle

## Abstract

In magnetic resonance imaging, precise measurements of longitudinal relaxation time (*T*_1_) is crucial to acquire useful information that is applicable to numerous clinical and neuroscience applications. In this work, we investigated the precision of *T*_1_ relaxation time as measured using the variable flip angle method with emphasis on the noise propagated from radiofrequency transmit field ($B_{1}^{+}$) measurements. The analytical solution for *T*_1_ precision was derived by standard error propagation methods incorporating the noise from the three input sources: two spoiled gradient echo (SPGR) images and a $B_{1}^{+}$ map. Repeated *in vivo* experiments were performed to estimate the total variance in *T*_1_ maps and we compared these experimentally obtained values with the theoretical predictions to validate the established theoretical framework. Both the analytical and experimental results showed that variance in the $B_{1}^{+}$ map propagated comparable noise levels into the *T*_1_ maps as either of the two SPGR images. Improving precision of the $B_{1}^{+}$ measurements significantly reduced the variance in the estimated *T*_1_ map. The variance estimated from the repeatedly measured *in vivo* *T*_1_ maps agreed well with the theoretically-calculated variance in *T*_1_ estimates, thus validating the analytical framework for realistic *in vivo* experiments. We concluded that for *T*_1_ mapping experiments, the error propagated from the $B_{1}^{+}$ map must be considered. Optimizing the SPGR signals while neglecting to improve the precision of the $B_{1}^{+}$ map may result in grossly overestimating the precision of the estimated *T*_1_ values.

## Introduction

Measurement of the longitudinal relaxation time (*T*_1_) of a sample is of paramount importance as evidenced by the fact that methods for its measurement appeared soon after the invention of NMR (Drain, 1949; Hahn, 1949). In MRI *T*_1_ mapping is widely used because it provides insight into the microstructure of brain tissue (Harkins et al., 2016) and can act as a biomarker of myelination (Dick et al., 2012; Lutti et al., 2013; Sereno et al., 2013). Hence, numerous *T*_1_ mapping methods are available (Kingsley, 1999). Although, typically taken as the gold standard, the inversion recovery approach is very time consuming (Stikov et al., 2015). Instead, the combination of multiple three dimensional (3D) spoiled gradient echo (SPGR) (Haase et al., 1986) images with short repetition times, variable flip angles (VFA) (Christensen et al., 1974; Fram et al., 1987) and appropriate spoiling (Zur et al., 1991; Ganter, 2006) offers a means of obtaining whole brain *T*_1_ maps in clinically feasible times (Deoni et al., 2005; Helms et al., 2008).

Several factors affect the accuracy and/or precision of *T*_1_ measurements obtained via the VFA method (Wang et al., 1987; Deoni et al., 2004; Preibisch and Deichmann, 2009; Schabel and Morrell, 2009; Helms et al., 2011; Wood, 2015). In particular, the bias introduced by the spatial inhomogeneity of the radiofrequency (RF) transmit field ($B_{1}^{+}$) is a well-known source of error (Stikov et al., 2015). Numerous methods exist for obtaining a $B_{1}^{+}$ map (Insko and Bolinger, 1993; Cunningham et al., 2006; Jiru and Klose, 2006; Dowell and Tofts, 2007; Yarnykh, 2007; Lutti et al., 2010; Sacolick et al., 2010; Nehrke and Börnert, 2012) and incorporating this into the *T*_1_ mapping pipeline has been shown to improve the accuracy of the estimated value of the *T*_1_ relaxation times (Venkatesan et al., 1998; Deoni, 2007; Helms et al., 2008; Lutti et al., 2013; Liberman et al., 2014). However, the precision of the $B_{1}^{+}$ map and how this diminishes the precision of the estimated *T*_1_ values has not been thoroughly addressed, especially not *in vivo*. Recently, a systematic comparison of the precision of different $B_{1}^{+}$ mapping methods was performed by Pohmann and Scheffler (2013). They reported the uncertainty in the measurements of the $B_{1}^{+}$ maps and found that the error could be up to approximately 30% for 3D variants. The results of their simulations and phantom experiments agreed well, but they did not investigate the precision of the $B_{1}^{+}$ mapping methods *in vivo* (expected to produce higher uncertainty) nor its impact on the estimated *T*_1_ relaxation times.

To further understand and quantify the effect of uncertainty (i.e., random variability) in $B_{1}^{+}$ maps on the precision of *T*_1_ mapping, a theoretical framework that can be applied *in vivo* and that considers the measurement uncertainty not only in the SPGR signals but also in the $B_{1}^{+}$ maps is needed. Hence the aims of this paper are:

- To theoretically investigate, within the clinically-feasible VFA approach, the propagation of noise from $B_{1}^{+}$ measurements to the estimated *T*_1_ values and compare this to the error propagated from the SPGR data.
- To verify that these theoretical estimates are valid for *in vivo* neuroimaging experiments.
- To show that decreasing the variability in $B_{1}^{+}$ measurements can dramatically increase the precision of estimated *T*_1_ values.

## Materials and methods

### Theory

**Figure 1:**
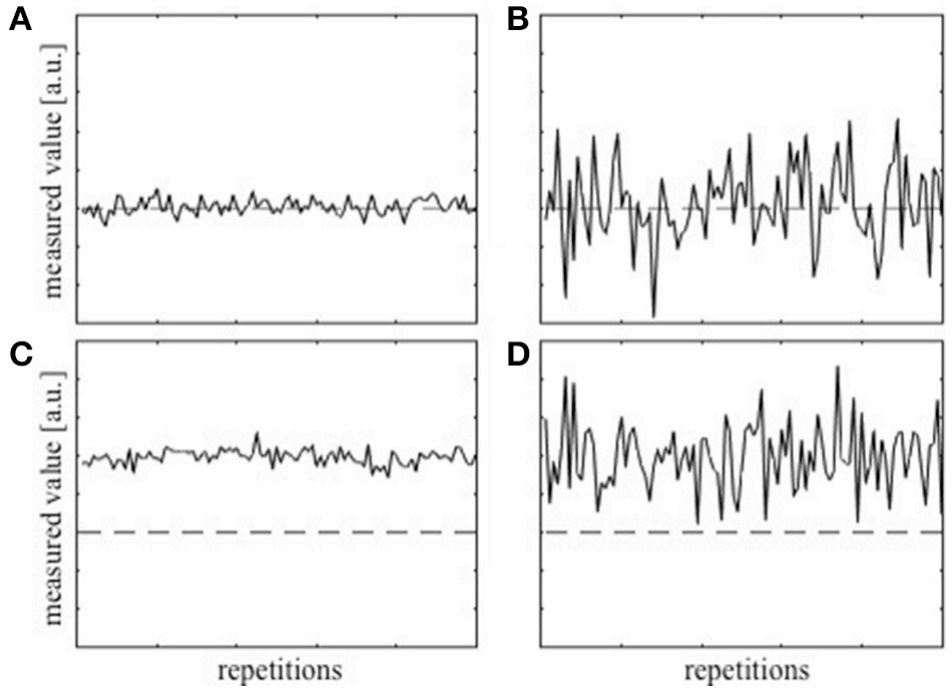
**Simulation of repeatedly measured data with (A)** high accuracy and high precision, **(B)** high accuracy, but low precision, **(C)** low accuracy and high precision, and **(D)** low accuracy and low precision. The dashed lines represent the true value.

Before proceeding with the theoretical framework for analyzing *T*_1_ precision, two terms, accuracy and precision, have to be defined clearly. Accuracy represents how close, on average, the measured value is to the true value and is often dependent on the level of systematic error present in the measurement. The deviation of the average measured value from the true value due to the systematic error is termed bias. On the other hand, precision represents how close the values from the repeated measurements are to each other and will depend on multiple factors, e.g., the sensitivity of the measurement device. Thus, the precision is a measure of uncertainty in the measurement irrespective of the true value. Figure 1A shows examples of measurements that are both accurate and precise, which is the target measurement scenario. Measurements can also be accurate but imprecise (Figure 1B), inaccurate but precise (Figure 1C), and neither accurate nor precise (Figure 1D). To collect a single data point with the hope that it is close to the true value, both accuracy and precision are important.

If the transverse magnetization is adequately spoiled before each RF pulse the SPGR signal amplitude is a function of *T*_1_, equilibrium magnetization (*M*_0_), effective transverse relaxation time ($T_{2}^{*}$), and imaging parameters, i.e., the repetition time (TR), flip angle α and echo time (TE) (Fram et al., 1987)

(1) $$\begin{array}{r} S=Asin\left(\alpha \right)\frac{1\;-\;exp\left(-\text{TR}/T_{1}\right)}{1\;-\;cos\left(\alpha \right)\cdot exp\left(-\text{TR}/T_{1}\right)} \end{array}$$

where $A=S_{0}exp\left(-\text{TE}/T_{2}^{*}\right)$. Here *S*_0_ is defined as *M*_0_ multiplied by the receive gain of the system and the receive coil sensitivity. Recently, rational approximation of the SPGR signal for small flip angles and short TR was suggested, which provides a simpler form of Equation (1) (Helms et al., 2008),

(2) $$\begin{array}{r} S≅A\alpha \frac{\text{TR}/T_{1}}{\alpha^{2}/2+\text{TR}/T_{1}} \end{array}$$

By acquiring two SPGR signals, *S*_1_ and *S*_2_, at two different flip angles, α_1_ and α_2_, *T*_1_ estimates can be obtained with a simple algebraic expression (Helms et al., 2008),

(3) $$\begin{array}{r} T_{1}=2\text{TR}\frac{S_{1}/\alpha_{1}\;-\;S_{2}/\alpha_{2}}{S_{2}\alpha_{2}\;-\;S_{1}\alpha_{1}} \end{array}$$

Since the VFA method relies on the flip angle dependency of the SPGR signal for *T*_1_ estimation, a correction for $B_{1}^{+}$ inhomogeneities is necessary in order to obtain unbiased *T*_1_ estimates. The spatially dependent $B_{1}^{+}$ correction factor, denoted as *f*_*B*1_, can be determined by normalizing the $B_{1}^{+}$ map such that 1 is the nominal flip angle. By multiplying α_1_ and α_2_ by *f*_*B*1_ in Equation (3) the $B_{1}^{+}$ bias corrected *T*_1_ equation can be obtained (Helms et al., 2008),

(4) $$\begin{array}{r} T_{1}=2\text{TR}\frac{S_{1}/\alpha_{1}\;-\;S_{2}/\alpha_{2}}{S_{2}\alpha_{2}\;-\;S_{1}\alpha_{1}}\frac{1}{f_{B1}^{2}} \end{array}$$

Measurement of a $B_{1}^{+}$ map and inclusion of the correction factor, *f*_*B*1_, in Equation [4] is intended to ensure the accuracy of the *T*_1_ estimates (Stikov et al., 2015). This correction is assumed to correspond to going from Figure 1C to Figure 1A. However, unless the precision of the $B_{1}^{+}$ map is high, the actual correction may correspond to going from Figure 1D to Figure 1B, or worse going from Figure 1C to Figure 1B thereby lowering the precision of the *T*_1_ estimate.

In the general VFA case, an expression for the variance of the estimated *T*_1_ ($\sigma_{T_{1}}^{2}$) can be calculated for a set of SPGR signals (*S*_1_, *S*_2_, …, and *S*_*N*_) measured with *N* different flip angles and a $B_{1}^{+}$ map (*f*_*B*1_) (Bevington and Robinson, 2003). Assuming statistically independent measurements of each signal the variance in the *T*_1_ estimate is

(5) $$\begin{array}{r} \sigma_{T_{1}}^{2}=\overset{N}{\underset{i=1}{\sum }}\left(\sigma_{S_{i}}^{2}{\left(\frac{\partial T_{1}}{\partial S_{i}}\right)}^{2}\right)+\;\sigma_{f_{B1}}^{2}{\left(\frac{\partial T_{1}}{\partial f_{B1}}\right)}^{2} \end{array}$$

where σ_*S*_*i*__ and σ_*f*_*B*1__ are the noise levels in *S*_*i*_ and *f*_*B*1_ respectively. For the VFA *T*_1_ mapping technique proposed by Helms et al. (2008) only two SPGR signals are acquired (*N* = 2) and *T*_1_ is calculated from Equation (4). Hence the variance of the estimated *T*_1_ propagated from a $B_{1}^{+}$ map expressed by the second term in Equation (5) is determined by the noise in a $B_{1}^{+}$ map (σ_*f*_*B*1__) and the partial derivative term which can be obtained from Equation (4):

(6) $$\begin{array}{r} \frac{\partial T_{1}}{\partial f_{B1}}=\frac{4\text{TR}}{f_{B1}^{3}}\cdot \frac{S_{2}\alpha_{1}\;-\;S_{1}\alpha_{2}}{a_{1}\alpha_{2}\left(S_{2}\alpha_{2}\;-\;S_{1}\alpha_{1}\right)} \end{array}$$

The *T*_1_ variance propagated from the two SPGR signals can be determined in the same way by the partial derivative terms with respect to *S*_*i*_:

(7) $$\begin{array}{r} \frac{\partial T_{1}}{\partial S_{1}}=\frac{2\text{TR}}{f_{B1}^{2}}\cdot \frac{S_{2}\left(\alpha_{2}^{2}\;-\;\alpha_{1}^{2}\right)}{\alpha_{1}\alpha_{2}{\left(S_{2}\alpha_{2}\;-\;S_{1}\alpha_{1}\right)}^{2}} \end{array}$$

(8) $$\begin{array}{r} \frac{\partial T_{1}}{\partial S_{2}}=\frac{2\text{TR}}{f_{B1}^{2}}\cdot \frac{-\;S_{1}\left(\alpha_{2}^{2}\;-\;\alpha_{1}^{2}\right)}{\alpha_{1}\alpha_{2}{\left(S_{2}\alpha_{2}\;-\;S_{1}\alpha_{1}\right)}^{2}} \end{array}$$

Each partial derivative term in Equations (6–8) is a weighting factor for the noise in the corresponding input signal (i.e., *S*_1_, *S*_2_, and *f*_*B*1_) in Equation (5).

### MR data collection

Data were collected on four adult volunteers using a 3T MRI scanner (Achieva Platform, Philips Healthcare, Best, The Netherlands). Four different experiments were performed. The first two experiments involved all four volunteers and the input variable *f*_*B*1_ was repeatedly measured either with small (**Experiment 1**) or with large spoiler gradients (**Experiment 2**) to assess two different levels of variance in the $B_{1}^{+}$ measurements. On one of the volunteers, the other two input variables, *S*_1_ and *S*_2_, were also repeatedly measured (**Experiments 3** and **4** respectively) to assess their variances and compare them with the variance introduced by the $B_{1}^{+}$ measurements.

Before each measurement the scanner performed a full preparatory phase of shimming, center frequency determination and RF transmit power calibration. The repeated measurements approach adopted here captured all noise sources, e.g., thermal/physiological noise, scanner stability, etc. In summary, the four different experiments were designed as follows:

**Experiment 1:** one *S*_1_, one *S*_2_, and six $B_{1}^{+}$ maps with small spoiler gradients.
**Experiment 2**: one *S*_1_, one *S*_2_, and six $B_{1}^{+}$ maps with large spoiler gradients.
**Experiment 3**: six *S*_1_, one *S*_2_, and one $B_{1}^{+}$ map with large spoiler gradients.
**Experiment 4**: one *S*_1_, six *S*_2_, and one $B_{1}^{+}$ map with large spoiler gradients.

The 3D SPGR sequence had 0.8 mm isotropic voxels, TR/TE1/TE2/TE3 = 25.0/4.6/11.5/18.3 ms, sensitivity encoding (SENSE) (Pruessmann et al., 1999) factor = 2.0, and scan time = 11.6 min. The SPGR images acquired at three different echo times were averaged to increase the signal-to-noise ratio (SNR) (Helms et al., 2008). The *S*_1_ and *S*_2_ images were acquired with the nominal flip angles of α_1_ = 6° and α_2_ = 20° respectively, resulting in images with predominantly proton-density (PD) weighting or *T*_1_ weighting. The $B_{1}^{+}$ maps were acquired at 4.0 mm isotropic resolution using the actual flip angle imaging (AFI) method (Yarnykh, 2007) with either small (*A*_G1_/*A*_G2_=45.33/761.2 mT.ms/m and TR1/TR2/TE = 20/100/2.2 ms) or large (*A*_*G*1_/*A*_*G*2_=931.8/1971.0 mT.ms/m, TR1/TR2/TE = 46/138/2.2 ms) spoiler gradients. *A*_G1_ and *A*_G2_ are the spoiler gradient areas on one axis for the interleaved acquisitions with TR1 and TR2, respectively. A nominal flip angle of 60° was used for this AFI $B_{1}^{+}$ map. To match the scan time (5.2 min) of the AFI acquisitions, the protocol using large spoilers also used a SENSE factor of 1.7. The six repetitions were chosen with consideration of the subject's ability to stay still during the measurements. To ascertain that six repetitions were adequate for a reliable estimate of the variability of input signals, variance was also calculated from the first three, first four and first five repetitions separately. The variance distribution converged after five measurements indicating that the estimate was stable and valid (data not shown).

### Data analysis

All images (*S*_1_, *S*_2_, and the $B_{1}^{+}$ map) were aligned to the first PD weighted image (i.e., the first of the six *S*_1_ in **Experiment 3**) using rigid body registration as implemented in SPM8 (Wellcome Trust Centre for Neuroimaging, UCL, UK). The $B_{1}^{+}$ maps were aligned by using the transformation matrix obtained in the alignment of the corresponding short TR AFI image to the first PD weighted image. Data were evaluated in two different ways to compare the *T*_1_ variances estimated from the *in vivo* measurements and the theoretical framework:

**Experimental variance evaluation**: Using Equation (4) six *T*_1_ maps were calculated for each of **Experiments 1–4**. The voxel-wise variance across these six *T*_1_ maps was then calculated. With this approach, the experimental noise level in the *T*_1_ map, σ_*T*_1_,exp_, was obtained.
**Theoretical variance evaluation**: The voxel-wise variance of the repeated *S*_1_, *S*_2_, or $B_{1}^{+}$ scans (i.e.,$\;\sigma_{S_{1}}^{2}$, $\sigma_{S_{2}}^{2}$, or $\sigma_{f_{B1}}^{2}$) was calculated and inserted into the theoretical noise propagation framework [Equations (5–8)], while assuming zero variance for the other two input signals. For example, in **Experiment 1** we assumed $\sigma_{S_{1}}^{2}=\sigma_{S_{2}}^{2}=0$ and evaluated $\sigma_{T_{1}}^{2}$ by multiplying $\sigma_{f_{B1}}^{2}$ (obtained from the repeated *in vivo* experiments) by the square of the expression given in Equation (6). $\sigma_{T_{1}}^{2}$ was similarly evaluated for **Experiments 2–4**. σ_*T*_1__ calculated by this approach is the theoretically-predicted voxel-wise noise level in the *T*_1_ map and is denoted by σ_*T*_1_,theo_.

Subsequently, coefficient of variation (*CV* = 100 × standard deviation / mean) maps were calculated to ease comparison of results across **Experiments 1–4**. Note that *CV* is inversely proportional to SNR.

The PD weighted image (*S*_1_) was segmented using SPM8 to extract the gray matter (GM) and white matter (WM) segments, which were subsequently thresholded at 0.9 (i.e., 90% probability of belonging to the respective tissue types). The resulting GM and WM masks were used to extract voxel-wise values from the three input images, the *T*_1_ maps and the corresponding *CV* maps. The median and interquartile range (IQR) of the *CV* values were then calculated for each tissue type independently.

## Results

**Figure 2:**
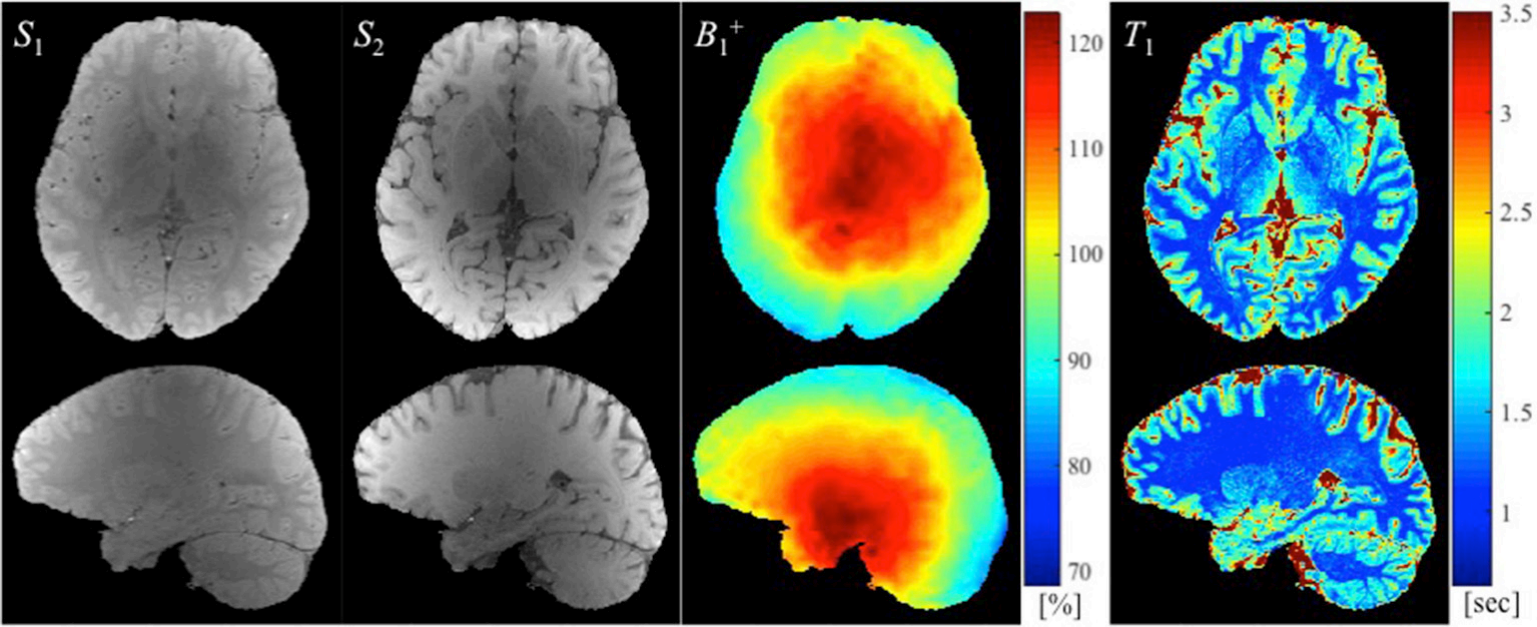
**The SPGR images with two different flip angles (***S***_**1**_ and ***S***_**2**_ in arbitrary units), the $B_{1}^{+}$ map (in the percentage of nominal flip angle), and the estimated ***T***_**1**_ map (in seconds) are shown from left to right**.

Example images used to calculate *T*_1_ maps in the work described here, namely two SPGR images (*S*_1_ and *S*_2_) and a $B_{1}^{+}$ map (in this case with large spoiler gradients) are shown in Figure 2 along with the resulting *T*_1_ map.

**Figure 3:**
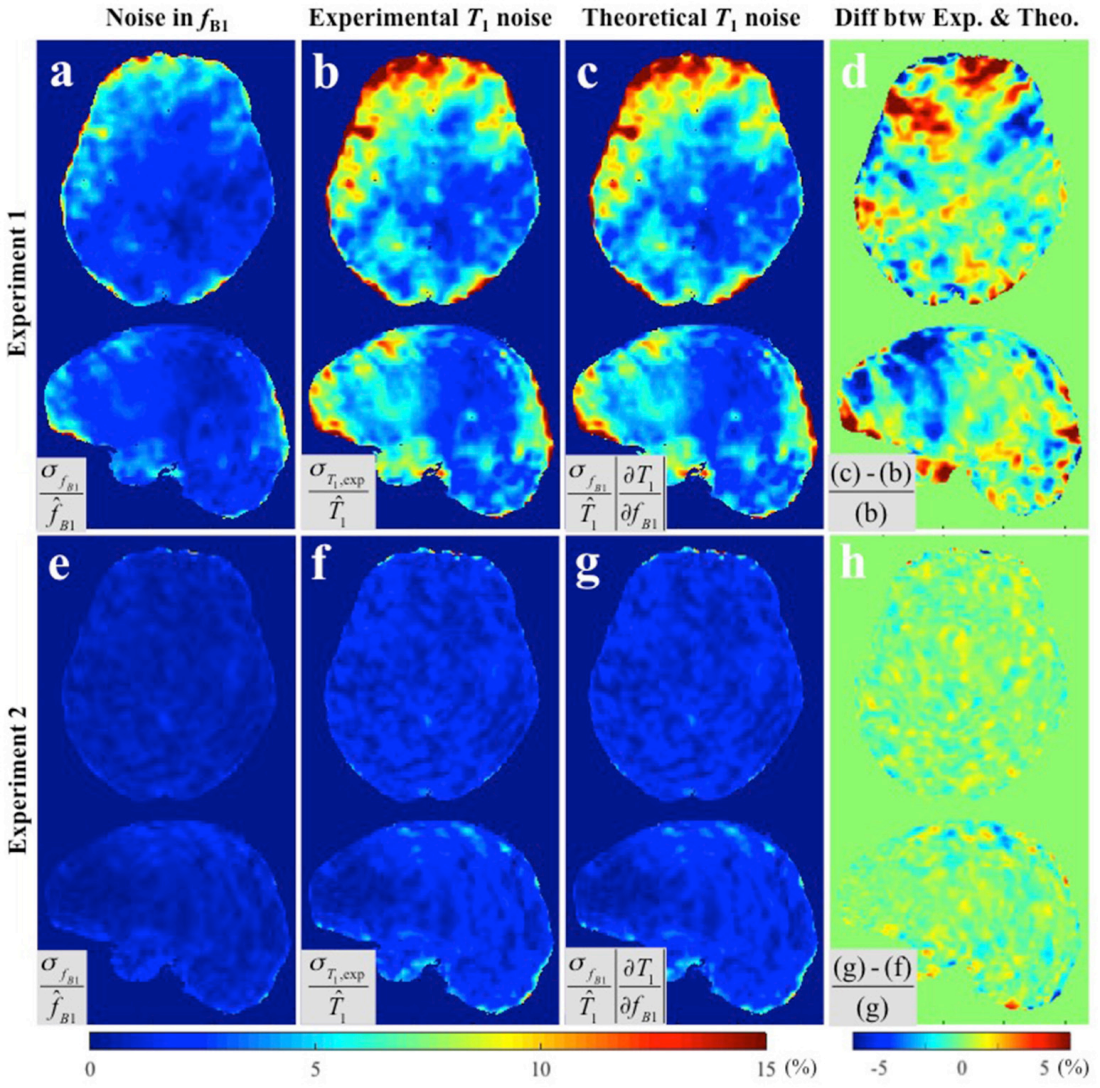
***CV*** **maps of (a,e)** the six *f*_*B*1_ acquired with small and large spoiler gradients in **Experiments 1** and **2**, **(b,f)** the noise of the measured *T*_1_ maps (**Experimental variance evaluation**), and **(c,g)** the theoretically-predicted noise in the *T*_1_ map from the second term in Equation (5), i.e., $100\cdot \sigma_{f_{B1}}\cdot \left(\partial T_{1}/\partial f_{B1}\right)/{\hat{T}}_{1}$ (**Theoretical variance evaluation**). **(d,h)** The percentage difference maps between experimentally-measured and theoretically-predicted noise in *T*_1_ estimates. All *CV* maps shown were calculated by the equations shown in the corresponding gray boxes multiplied by 100. Here, ${\hat{f}}_{B1}$ and ${\hat{T}}_{1}$ represent the means across six *f*_*B*1_ and corresponding *T*_1_ maps respectively.

Figure 3 provides the results of both methods of variance estimation for **Experiments 1–2**. Column 1 shows the *CV* maps calculated from the variance across the repeated $B_{1}^{+}$ measurements, i.e., $\sigma_{f_{B1}}^{2}$, with small (Figure 3a) and large (Figure 3e) spoiler gradients. The experimental and theoretical evaluations of the noise level in the *T*_1_ map propagated from the variance in the $B_{1}^{+}$ map (i.e., σ_*T*_1_,exp_ and σ_*T*_1_,theo_) are shown in column 2 and 3 respectively. Increased spoiling resulted in improved precision of the $B_{1}^{+}$ maps (compare Figure 3a with Figure 3e), which in turn reduced the variance of the *T*_1_ estimates both experimentally and theoretically (compare Figure 3b with Figure 3f and Figure 3c with Figure 3g). Column 4 shows the percentage difference map between the experimentally-measured (σ_*T*_1_,exp_) and the theoretically-predicted (σ_*T*_1_,theo_) noise levels in *T*_1_. In general the discrepancy between σ_*T*_1_,exp_ and σ_*T*_1_,theo_ was small. For **Experiment 1** (small spoiler), the mean discrepancies (average of absolute percentage difference values) were 1.97 and 1.44% in GM and WM respectively. For **Experiment 2** (large spoiler) these mean discrepancies were reduced to 0.52 and 0.46% respectively. The discrepancy maps (Figures 3a,h) show that, if the noise in the input signal is small, the theoretical prediction works better. This is expected from Equation (5).

**Figure 4:**
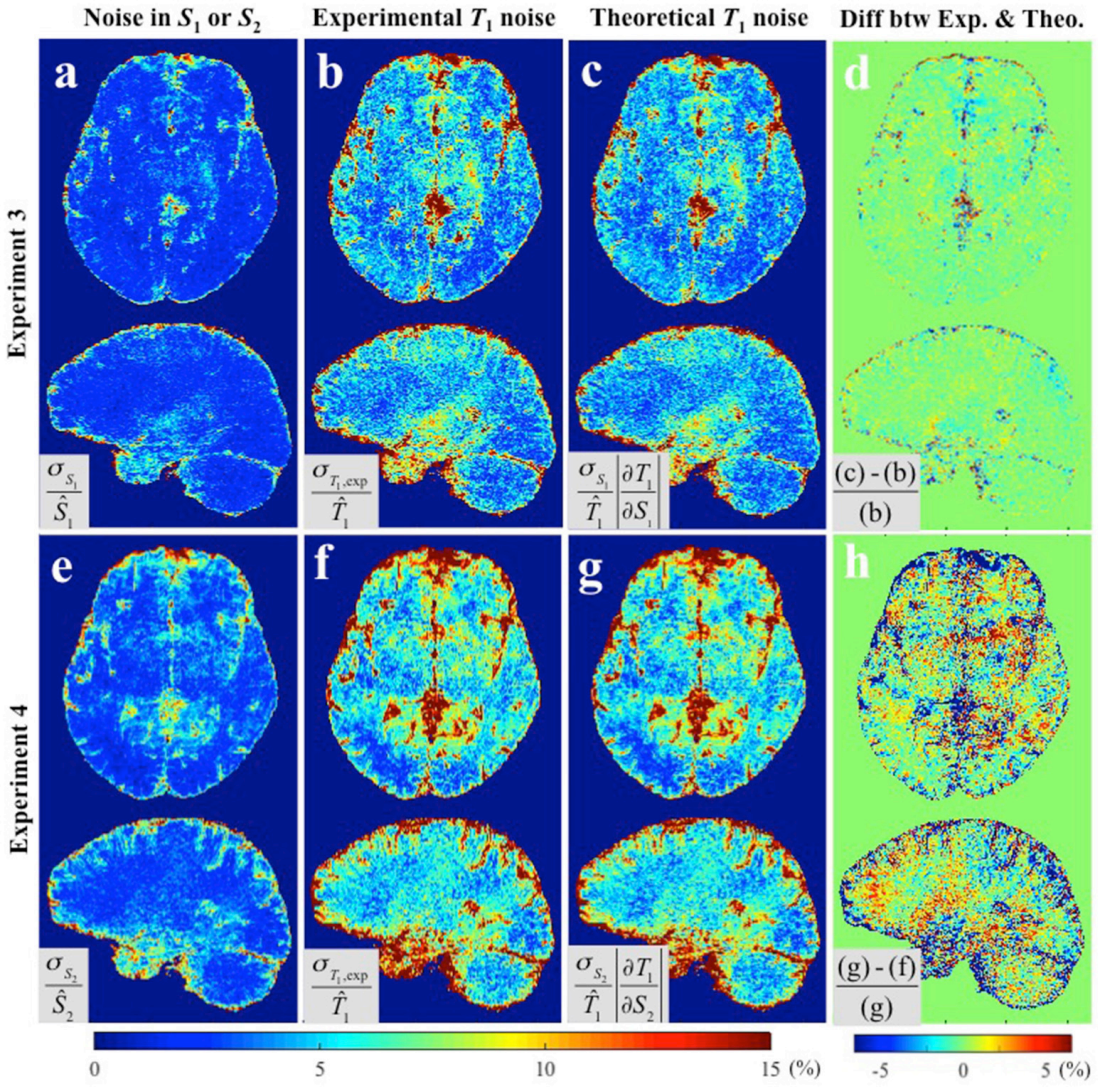
***CV*** **maps (a,e)** of the input images in **Experiments 3** and **4** (*S*_1_ and *S*_2_ respectively), **(b,f)** the experimentally obtained variability in the estimated *T*_1_ maps using **Experimental variance evaluation**, and **(c,g)** the theoretically-predicted *T*_1_ noise from the first term in Equation (5), i.e., $100\cdot \sigma_{S_{1}}\cdot \left(\partial T_{1}/\partial S_{1}\right)/{\hat{T}}_{1}$ and $100\cdot \sigma_{S_{2}}\cdot \left(\partial T_{1}/\partial S_{2}\right)/{\hat{T}}_{1}$ respectively (**Theoretical variance evaluation**). **(d)** The percentage difference between **(b)** and **(c)**. **(h)** The percentage difference between **(f)** and **(g)**. Results are shown separately for **Experiment 3 (a–d)** and **Experiment 4 (d–h)**. All *CV* maps shown were calculated by the equations shown in the corresponding gray boxes multiplied by 100. Ŝ_1_, Ŝ_2_, and ${\hat{T}}_{1}$ in gray boxes denote the average values of six *S*_1_, *S*_2_, and corresponding *T*_1_ maps respectively.

Figure 4 shows the results of **Experiments 3** and **4** where *S*_1_ and *S*_2_ were measured repeatedly as a comparison to the repeated acquisitions of the $B_{1}^{+}$ maps. Figure 4a,e show the *CV* maps across the repeated measurements of *S*_1_ and *S*_2_ (i.e., *PD*-weighted and *T*_1_-weighted signal respectively). The results of **Experimental and Theoretical variance evaluations** and the percentage difference map between them are shown in Figures 4b–d,f–h respectively. The mean discrepancies between σ_*T*_1_,exp_ and σ_*T*_1_,theo_ in GM/WM were 0.62/0.37% and 3.03/1.73% for **Experiments 3** and **4** respectively. The larger discrepancy between theory and experiment in **Experiment 4** (Figure 4h) compared to **Experiment 3** (Figure 4d) could be attributed to the larger input noise in the repeated *S*_2_ measurements (Figure 4e) than in the repeated *S*_1_ measurements (Figure 4a). Nonetheless, the fact that the overall discrepancies are small (column 4 of Figures 3, 4) demonstrates the validity of the theoretical framework presented in Equations (5–8) for estimating the variance in *T*_1_ maps measured *in vivo*.

**Figure 5:**
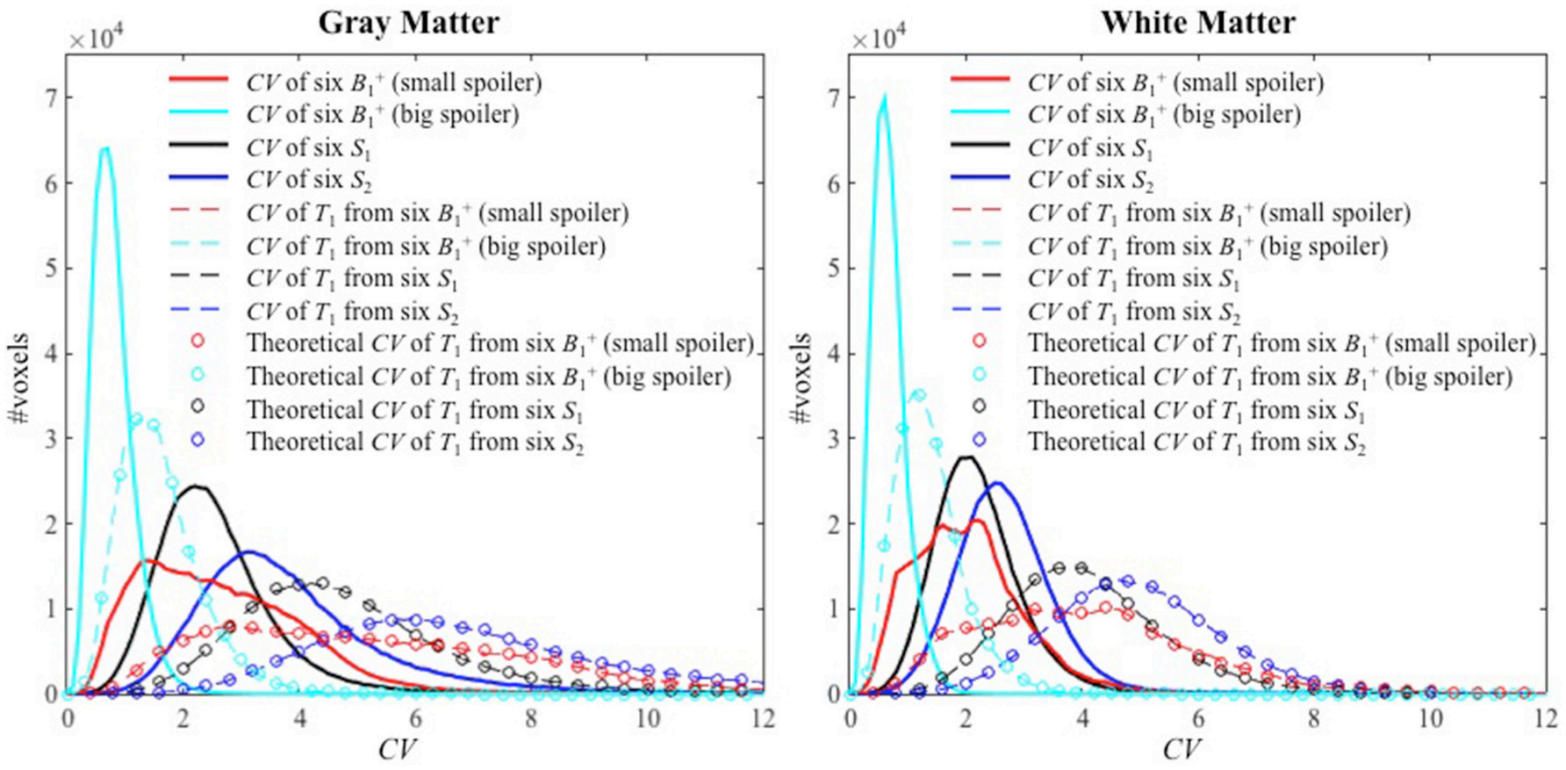
**The histograms of ***CV*** values for Experiments 1–4 inside GM (left) and WM (right)**. The solid lines, dashed lines, and circles represent the *CV* of the six input images, the experimental *CV* of *T*_1_ calculated from the six input images (**Experimental variance evaluation**), and the theoretically-predicted *CV* of *T*_1_ (**Theoretical variance evaluation**) respectively. The histograms for **Experiments 1–4** are shown with red, cyan, black and blue colors, respectively.

Histograms of the *CV* maps for GM and WM are shown in Figure 5 from the voxel-wise variance in the input images (solid lines), *CV* maps obtained with **Experimental variance evaluation** (dashed lines) or **Theoretical variance evaluation** (circles) for **Experiments 1–4**. The histogram of the theoretically-predicted *CV* values agreed well with that of the experimentally-calculated *CV* values. Note that the histograms for **Experiment 2** (cyan) shifted toward lower *CV* values and sharpened significantly compared to those for **Experiment 1** (red), indicating that the *T*_1_ precision was greatly improved by the increased spoiler gradients, across the entire brain. Except for the distributions from **Experiment 1** with small spoiler gradients (solid red) where neither of the distributions from GM nor WM is symmetric, the distributions of *CV* values in WM are closer to the normal distribution than those in GM.

Median and IQR values were used to quantitatively summarize the *CV* histograms given that they were not all normally distributed. Table 1 shows the results for **Experiments 1** and **2** on four different subjects and Table 2 for **Experiments 3** and **4** on one subject. These results indicate that the noise in *S*_1_, *S*_2_, and *f*_*B*1_ propagated similarly into the *T*_1_ maps, such that the *CV* approximately doubled between the input signals and the calculated *T*_1_ maps. Also, the *CV* values of the *T*_1_ maps calculated via **Experimental** and **Theoretical variance evaluations** were similar in both GM and WM. As noted previously, the *CV* values decreased dramatically going from **Experiment 1** with small spoiler to **Experiment 2** with large spoiler. This was the case for subjects 1–3 (see Table 1). For subject 4, however, the *CV* values did not decrease (gray cell background in Table 1). This was likely due to instability in the RF transmit chain and it demonstrates the validity of the theoretical framework for different sources of error.

**Table 1:** **Median and (IQR) of ***CV*** values inside GM and WM for Experiments 1–2**.

		**Experiment 1 (six** $B_{1}^{+}$ **w/small spoiler)**		**Experiment 2 (six** $B_{1}^{+}$ **w/big spoiler)**	
		**GM**	**WM**	**GM**	**WM**
Subject 1	*CV* of repeated $B_{1}^{+}$ measurements	2.46 (1.99)	1.95 (1.23)	0.75 (0.44)	0.64 (0.37)
	Experimental *CV* of *T*_1_	4.93 (4.04)	3.92 (2.52)	1.49 (0.88)	1.28 (0.73)
	Theoretical *CV* of *T*_1_	4.90 (3.96)	3.89 (2.46)	1.49 (0.88)	1.28 (0.73)
Subject 2	*CV* of repeated $B_{1}^{+}$ measurements	2.67 (1.72)	1.77 (1.13)	0.70 (0.37)	0.59 (0.29)
	Experimental *CV* of *T*_1_	5.37 (3.41)	3.54 (2.29)	1.39 (0.74)	1.19 (0.58)
	Theoretical *CV* of *T*_1_	5.34 (3.43)	3.53 (2.26)	1.39 (0.74)	1.19 (0.58)
Subject 3	*CV* of repeated $B_{1}^{+}$ measurements	3.70 (2.18)	2.74 (1.32)	0.79 (0.42)	0.64 (0.35)
	Experimental *CV* of *T*_1_	7.48 (4.62)	5.44 (2.71)	1.57 (0.84)	1.28 (0.69)
	Theoretical *CV* of *T*_1_	7.38 (4.30)	5.46 (2.63)	1.57 (0.84)	1.28 (0.69)
Subject 4	*CV* of repeated $B_{1}^{+}$ measurements	2.91 (1.52)	2.11 (1.00)	3.30 (0.59)	3.28 (0.47)
	Experimental *CV* of *T*_1_	5.81 (3.04)	4.21 (1.97)	6.77 (1.25)	6.75 (1.00)
	Theoretical *CV* of *T*_1_	5.82 (3.03)	4.22 (1.98)	6.57 (1.17)	6.54 (0.93)

*IQR, interquartile range; CV, coefficient of variation; GM, gray matter; WM, white matter*.

*Subject 4 had high variability in f_B1_ and T_1_ (gray shade values) even in Experiment 2, which may be due to hardware instability in the RF transmit chain*.

**Table 2:** **Median and IQR of ***CV*** values inside GM and WM for Experiments 3–4**.

		**Experiment 3 (six** ***S*****1)**		**Experiment 4 (six** ***S*****2)**	
		**GM**	**WM**	**GM**	**WM**
Subject 1	*CV* of repeated *S*_1_ or *S*_2_ measurements	2.41 (1.17)	2.12 (0.92)	3.56 (1.83)	2.61 (1.03)
	Experimental *CV* of *T*_1_	4.56 (2.23)	3.98 (1.73)	6.69 (3.58)	4.90 (1.94)
	Theoretical *CV* of *T*_1_	4.55 (2.23)	3.98 (1.73)	6.69 (3.49)	4.90 (1.92)

*IQR, interquartile range; CV, coefficient of variation; GM, gray matter; WM, white matter*.

## Discussion

Using the VFA method, the precision of *T*_1_ relaxation time measurements depends not only on the SNR of the SPGR images but, crucially, also on the error propagated from the $B_{1}^{+}$ map that is used to correct the bias caused by spatial inhomogeneity in the achieved flip angle. The precision of the $B_{1}^{+}$ map is often overlooked as a source of uncertainty in *T*_1_ measurements. Here we have derived analytical solutions for the error propagated to the *T*_1_ relaxation time estimates (Equations 5–8). This analysis indicates that the three signal sources (the two SPGR images with different flip angles and the $B_{1}^{+}$ map) propagate noise into the *T*_1_ estimates to approximately the same degree, with the *CV* approximately doubling between each of the three signal sources and the *T*_1_ estimate (Tables 1, 2). By examining two distinct noise levels in the *B*_1_^+^ maps (by manipulating the degree of spoiling), we could show that the precision of the *T*_1_ map can be greatly improved by increasing the precision of the $B_{1}^{+}$ mapping procedure.

We have experimentally validated the analytical framework by performing repeated experiments to estimate the voxel-wise variance of the *T*_1_ maps. We found overall agreement between the theoretical predictions (σ_*T*_1_,theo_) and the experimental measures (σ_*T*_1_,exp_), especially when the input noise, and accordingly *T*_1_ noise, is small as in **Experiments 2** and **3**. For example, Figure 4d shows discrepancy of less than 1% inside GM and WM and relatively high discrepancy only in voxels containing cerebrospinal fluid (CSF), which we attribute to the fact that CSF has a significantly longer *T*_1_ than GM and WM, for which the VFA sequence was optimized. With higher input noise the discrepancy between theory and measurement tended to increase (Figures 3d, 4h). This is because Equation (5) predicts the propagated error correctly only when σ_*S*_*i*__ and σ_*f*_*B*1__ are small enough that the constant slope approximation (i.e., constant partial derivative) is valid over the ranges of σ_*S*_*i*__ and σ_*f*_*B*1__ in the *S*_*i*_/*f*_*B*1_ vs. *T*_1_ graph. Nonetheless, in the range of experimentally measured noise from our *in vivo* experiments, the discrepancies were small inside both GM and WM.

Subject 4 had high variability in *f*_*B*1_ and therefore in the estimated *T*_1_, even in **Experiment 2**, which used big spoiler gradients to minimize the variance. This may be due to one of the parameters associated with the determination of the RF transmit voltage, which were observed to fluctuate more across the six $B_{1}^{+}$ acquisitions in subject 4 than across the acquisitions from the other three subjects. This fluctuation might be due to hardware instability in the RF transmit chain. The necessity to keep the RF transmit voltage constant for reliable quantification of *T*_1_ has been reported previously in Lutti and Weiskopf (2013). Therefore, it is reasonable to consider the RF transmit instability as a reason behind the reduced precision of the repeated $B_{1}^{+}$ map acquisitions in this case. This observation demonstrates that not only the acquisition method, e.g., degree of spoiling used, but also the hardware settings need to be considered when optimizing the precision of $B_{1}^{+}$, and by extension *T*_1_, measurements.

The *CV* maps from **Experiment 1** had asymmetric left-right distributions as shown in Figures 3a–c for subject 1. While three out of four subjects manifested asymmetric distributions, one of them showed a strong pattern of left-right symmetry (data not shown), indicating that the spatial distribution of precision is subject-specific. This may be due to susceptibility effects from the air-tissue interface, how well the shimming procedure can correct for local field distortions, positioning of the subject in the scanner and interaction with the transmit field. We also found that the histograms of *CV* values in GM did not tend to be normally distributed compared to those in WM. This may be due to the fact that more GM voxels suffer partial volume effects with CSF and the VFA acquisition was optimized for the *T*_1_ values of GM/WM not the significantly longer *T*_1_ of CSF. The non-normal distributions in the histograms from **Experiment 1** for both GM and WM show that the $B_{1}^{+}$ maps with small spoiler gradients are dominated by noise sources other than thermal noise.

**Figure 6:**
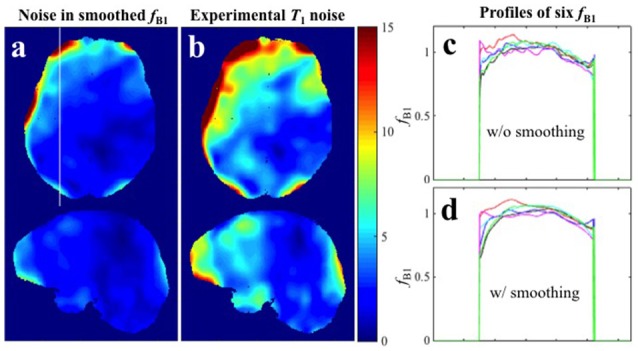
**Spatial smoothing effects in Experiment 1 with small spoiler gradients. (a)** *CV* map of the six *f*_*B*1_ after smoothing. **(b)** *CV* map of the six *T*_1_ maps estimated with the six smoothed *f*_*B*1_ (**Experimental variance evaluation**). **(c,d)** Profiles along the white line in **(a)** for the six original *f*_*B*1_ maps and six smoothed *f*_*B*1_ maps respectively.

Because the transmit RF field map is smoothly varying, a commonly recommended practice for reducing noise in $B_{1}^{+}$ maps is spatial smoothing. It must be noted however that systematic offset in a given image cannot be corrected by spatial smoothing. As an example see Figure 6 where the 6 $B_{1}^{+}$ maps from **Experiment 1** were smoothed by a 3D Gaussian kernel with standard deviation of 4 × 4 × 4 mm^3^. It is evident from the profile extracted from the white line in Figure 6a that spatial smoothing of the 6 individual $B_{1}^{+}$ maps separately leaves a systematic offset uncorrected (Figures 6c,d). In such cases, when thermal noise does not dominate the error sources but the systematic offset is random in the different repetitions, a more appropriate procedure is averaging multiple acquisitions. Although, repeated measurement of the $B_{1}^{+}$ map requires additional time, it is usually still the more efficient way to proceed because high-resolution SPGR images take significantly longer to acquire (in our case more than twice as long). This recommendation is further supported by our finding that the $B_{1}^{+}$ maps propagate approximately the same error as either of the two SPGR images.

When optimizing *T*_1_ mapping protocols, previous work has focused mainly on optimizing acquisition parameter settings, most notably the flip angles used to acquire the SPGR images, that minimize uncertainty in the measured *T*_1_ value (Weiss et al., 1980; Wang et al., 1987; Schabel and Morrell, 2009; Helms et al., 2011; Wood, 2015). Although bias resulting from the spatial non-uniformity of the transmit RF transmit field is also well known and is commonly corrected by incorporating a $B_{1}^{+}$ map into the calculations (Helms et al., 2008; Yarnykh, 2010; Lutti et al., 2013; Stikov et al., 2015), the precision with which the $B_{1}^{+}$ map is obtained is typically ignored. Pohmann and Scheffler compared the precision of several $B_{1}^{+}$ mapping methods and found widely varying results depending on the method used and the nominal flip angle to be measured (Pohmann and Scheffler, 2013). Our results further demonstrate the necessity to consider the precision of $B_{1}^{+}$ mapping when using these to correct bias in the *T*_1_ relaxation time maps. Rather than the assumed high precision estimate of *T*_1_ (Figure 1A), one may arrive at a result that on the average is correct but may also have a high level of uncertainty (Figure 1B). This will have the greatest impact *in vivo* where there are more sources of noise (e.g., physiologically driven noise) and will reduce the detectable effect size in both cross-sectional and longitudinal studies in which *T*_1_ measurements are used as a biomarker (Lutti et al., 2013).

In this study we used the AFI method for $B_{1}^{+}$ mapping, which was proposed and optimized by Yarnykh (Yarnykh, 2007, 2010) and has been shown to perform comparatively well (Pohmann and Scheffler, 2013). From **Experiments 1–2**, we showed that increasing spoiler gradients makes the estimation of *T*_1_ not only more accurate as previously reported (Yarnykh, 2010) but also more precise due to the complete spoiling of the transverse magnetization. Pohmann and Scheffler (2013) found that for a 60° nominal flip angle their implementation of the AFI method had an uncertainty of 3° (5%) in simulations and 4° (7.5%) in phantom experiments. The *CV* of approximately 3% in the $B_{1}^{+}$ map that we observed *in vivo* (Tables 1, 2) is in line with these findings, and may even underestimate the $B_{1}^{+}$related variance that can be expected to propagate into common *T*_1_ mapping protocols from the map. However, the theoretical framework presented here makes no assumption on the choice of $B_{1}^{+}$ mapping approach. These findings are equally applicable, regardless of the $B_{1}^{+}$ mapping method used or how it was optimized to have high precision.

In both the *in vivo* experimental variance (**Experimental variance evaluation**) and the theoretical framework (**Theoretical variance evaluation**) we considered the noise propagating from the three input signals separately (Equations 5–8 and **Experiments 1–4**). This provides a convenient way in which to compare the effect of the three noise sources on the uncertainty of the final *T*_1_ map. In practice, however, the errors propagating from the three sources are summed (Equation 5). Although *CV*s for the averaged SPGR signals and the $B_{1}^{+}$ maps were similar in our *in vivo* experiments, the precision of $B_{1}^{+}$ maps can vary significantly depending on the $B_{1}^{+}$ mapping approach (see for example Figure 6 in Pohmann and Scheffler, 2013), which shows that in phantom measurements some of the $B_{1}^{+}$ mapping methods, especially the 2D variants, can suffer higher uncertainty for certain acquisition parameter sets). Therefore, neglecting the uncertainty in the $B_{1}^{+}$ map can lead to significant erroneous overestimation of the precision of the calculated *T*_1_ value.

Also note that including more than two SPGR images with more than two different flip angles for the *T*_1_ estimation may even lead to the increased variability in *T*_1_ since the noise from the additional SPGR images is additive to the total variance in *T*_1_ ($\sigma_{T_{1}}^{2}$) according to Equation (5). This is also reflected by the fact that previous evaluations of uncertainty within the VFA regime conclude that when acquiring additional images the optimal approach is to acquire at the same flip angle and average (Wang et al., 1987; Helms et al., 2011).

To assess the uncertainty in the measurements of SPGR images and $B_{1}^{+}$ maps we performed repeated *in vivo* experiments for each of the input images. In such an approach, the uncertainty in each variable includes all sources, e.g., thermal noise, scanner instability, scanner drift, physiological noise and the test/re-test variability (e.g., differences in the optimized shim currents, or power amplifier calibration etc.), whereas usually only the thermal noise components are considered (Cheng and Wright, 2006). Had we simply estimated the thermal noise component by extracting the standard deviation of pixels in the background the theoretically-predicted uncertainty (**Theoretical variance evaluation**) would have seriously underestimated the uncertainty found in the *in vivo* *T*_1_ relaxation time measurement (**Experimental variance evaluation**). In addition we would have confounds due to the Rician noise distribution of magnitude images and the image reconstruction scheme chosen (Constantinides et al., 1997).

A wide array of methods exists for measuring the *T*_1_ relaxation time (Kingsley, 1999). A recently proposed method (Helms et al., 2008) was chosen here because it has been broadly used (Dick et al., 2012; Sereno et al., 2013; Callaghan et al., 2014) and optimized (Helms et al., 2011). However, our findings regarding the dependence of the precision of the *T*_1_ measurement on the level of uncertainty in the $B_{1}^{+}$ map is not expected to be unique to this method of *T*_1_ relaxation time measurement.

We conclude that when estimating the uncertainty of *T*_1_ mapping methods, the error propagated from the $B_{1}^{+}$ map must also be considered. Optimizing the SPGR signals while neglecting to improve the precision of the $B_{1}^{+}$ map will result in a significant underestimation of the final uncertainty in the calculated *T*_1_ relaxation time. Maximizing the precision of the adopted $B_{1}^{+}$ mapping approach is crucial for studies using *T*_1_ as an imaging biomarker, which require high sensitivity (minimum variance), e.g., to investigate subtle differences in the micro-architectural organization of the brain.

## Ethics Statement

This study was carried out in accordance with the recommendations of The Kantonale Ethics Komitee (i.e., regional ethics committee) of Zurich with written informed consent from all subjects. The protocol was approved by The Kantonale Ethics Komitee.

## Author contributions

YL, MC, and ZN designed the study; YL and ZN acquired the data; MC provided software for data analysis; YL, MC, and ZN analyzed data, interpreted the results and wrote the manuscript.

### Conflict of interest statement

The authors declare that the research was conducted in the absence of any commercial or financial relationships that could be construed as a potential conflict of interest.
